# Lutein Leads to a Decrease of Factor D Secretion by Cultured Mature Human Adipocytes

**DOI:** 10.1155/2015/430741

**Published:** 2015-10-04

**Authors:** Yuan Tian, Aize Kijlstra, Johan Renes, Martin Wabitsch, Carroll A. B. Webers, Tos T. J. M. Berendschot

**Affiliations:** ^1^University Eye Clinic Maastricht, Postbus 5800, 6202 AZ Maastricht, Netherlands; ^2^Department of Human Biology, NUTRIM School for Nutrition, Toxicology and Metabolism, Maastricht University, Maastricht, Netherlands; ^3^Sektion Pädiatrische Endokrinologie u. Diabetologie, Universität Ulm, Germany

## Abstract

*Purpose*. Complement plays an important role in the pathogenesis of age related macular degeneration (AMD) and trials are currently being conducted to investigate the effect of complement inhibition on AMD progression. We previously found that the plasma level of factor D (FD), which is the rate limiting enzyme of the complement alternative pathway, was significantly decreased following lutein supplementation. FD is synthesized by adipose tissue, which is also the main storage site of lutein. In view of these findings we tested the hypothesis whether lutein could affect FD synthesis by adipocytes. *Methods*. A cell line of mature human adipocytes was incubated with 50 *μ*g/mL lutein for 24 and 48 h, whereafter FD mRNA and protein expression were measured. *Results*. Lutein significantly inhibited adipocyte FD mRNA expression and FD protein release into adipocyte culture supernatants. *Conclusions*. Our earlier observations showing that a daily lutein supplement in individuals with early signs of AMD lowered the level of circulating FD might be caused by blocking adipocyte FD production.

## 1. Introduction

Age related macular degeneration (AMD) is the leading cause of irreversible visual impairment among older adults in industrialized countries and is now recognized as the third cause of global blindness [1–3]. It is a multifactorial disease with age as the most important risk factor [4]. Modifiable factors like smoking and nutrition also play a role in the development of AMD [5–9]. Of the nutrient factors, the carotenoids lutein and zeaxanthin have been shown to be beneficial in the maintenance of proper vision [10–14]. Patients with AMD often exhibit lower dietary intake of lutein compared to control subjects [15–17]. Further, intervention studies have proven that intake of antioxidant supplements containing lutein can affect the progression to the advanced stages of AMD [10–13].

Lutein and zeaxanthin are the main constituents of the macular pigment [18, 19]. They absorb light between 390 and 540 nm [20–22], thereby shielding the retina from harmful blue light that causes photochemical light damage [23]. In addition, macular pigment is capable of scavenging free radicals [24] which also results in a protective antioxidant effect in the retina [18]. Recently it has been shown that lutein also has anti-inflammatory properties [25], having beneficial effects in various models of experimental ocular inflammation, such as endotoxin induced uveitis [26], retinal ischemia [27, 28], and diabetic retinopathy [29–31]. This may also have implications in AMD, due to an inflammatory mechanism involving the alternative complement pathway that has recently been implicated to play a major role in the pathogenesis of AMD [32].

Various complement proteins have been found to be associated with drusen [33]. These accumulations of extracellular material are located between Bruch's membrane and the retinal pigment epithelium of the eye and their presence is a common early sign of AMD. Genetic studies furthermore revealed that variants in the genes of complement factors, especially complement factor H (CFH), increased the susceptibility to AMD [34–36]. In addition, various complement activation products were increased in the circulation of AMD patients, providing evidence for a systemic inflammatory component to the disease pathogenesis [37–39].

Whether lutein administration could affect the inflammatory component of AMD is not clear yet. The first clues came from studies showing that administering lutein had a beneficial effect in an experimental model of AMD [40, 41]. Recently, we have reported that daily supplementation with lutein over a time period of twelve months led to a significant decrease in the plasma levels of the complement factors: factor D (FD), C3d, C5a, and sC5b-9 [42, 43].

The activation of the alternative complement pathway involves a number of cleavage reactions and amplification steps whereby complement components interact with each other in a strictly regulated manner. FD is a rate limiting enzyme in the activation sequence of the alternative pathway and as such a key player in complement homeostasis [44, 45]. FD is also known as adipsin, since its main source is adipose tissues, where it is secreted by mature adipocytes [46]. Adipose tissue is also a main storage site for carotenoids such as lutein and zeaxanthin [47–49]. Whether lutein influences FD secretion by adipose cells is unknown and was the subject of the study described here.

## 2. Methods

### 2.1. Cell Culture

Simpson-Golabi-Behmel syndrome (SGBS) preadipocytes were obtained from Professor Wabitsch (University of Ulm, Ulm, Germany) [50]. These cells originate from an adipose tissue specimen of an SGBS patient and have been used for a number of studies on adipose differentiation, adipocyte glucose uptake, lipolysis, apoptosis, regulation of expression of adipokines, and protein translocation [51]. SGBS preadipocytes at generation 20 were seeded in 25 cm^2^ culture flasks in DMEM/F12 (Catalog number 31330, Invitrogen) containing 1% penicillin/streptomycin (P/S, Invitrogen, Catalog number 15140-122), 3.3 mM biotin (Sigma-Aldrich, Catalog number B-4639), 1.7 mM D-pantothenic acid (Sigma-Aldrich, Catalog number P-5155), and 10% fetal calf serum (FCS) (Gibco Invitrogen, Breda, The Netherlands) and cultured for 6 days to reach 90% confluence. Cells were washed 3 times with phosphate buffered saline (PBS) and then changed to a serum- and albumin-free differentiation medium consisting of DMEM/F12 supplemented with 2 *μ*mol/L rosiglitazone (Cayman, Catalog number 71740), 25 nmol/L dexamethasone (Sigma-Aldrich, Catalog number D-1756), 0.5 mM methyl iso-buthylxantine (Sigma-Aldrich, Catalog number I-5879), 0.1 *μ*M cortisol (Sigma-Aldrich, Catalog number H-0888), 0.01 mg/mL human transferrin (Sigma-Aldrich, Catalog number T-2252), 0.2 nM triiodothyronine (T3, Sigma-Aldrich, Catalog number T-6397), and 20 nM human insulin (Sigma-Aldrich, Catalog number I-1507) at day 0. Medium was refreshed after two days and at day 4 the medium was changed and cells were further cultured in DMEM/F12 supplemented with 0.1 *μ*M cortisol (Sigma-Aldrich, Catalog number H-0888), 0.01 mg/mL human transferrin (Sigma-Aldrich, Catalog number T-2252), 0.2 nM triiodothyronine (T3, Sigma-Aldrich, Catalog number T-6397), and 20 nM human insulin (Sigma-Aldrich, Catalog number I-1507). Cells were incubated in this medium for several days and the culture medium was refreshed every two days. Small lipid droplets became visible after approximately 7 days. After 10 days, approximately 10% are differentiated and showed massive triglyceride accumulation. On culture day 20, the cells were filled with high amounts of intracellular lipids and the differentiation grade was approximately 50–60%. During the whole process, all cells were cultured under humidified atmosphere containing 5% CO_2_ at 37°C.

### 2.2. Experimental Protocol

To study the effect of lutein (Sigma, Switzerland, Catalog number X6250, purity > 99%), the mature adipocytes (for culture detail, see above) were washed three times with phosphate buffered saline (PBS) and then further incubated in serum-free DMEM/F12 medium for one day before treatment. The medium was supplemented with only transferrin, D-pantothenic acid, and biotin. Lutein was dissolved in dimethyl sulfoxide (DMSO, Sigma, Switzerland, Catalog number D8418, BioReagent, for molecular biology, purity ≥ 99.9%) and then serially diluted to the working concentrations with DMEM/F12 culture medium. As vehicle control we used the same medium with 0.5% DMSO solvent. The lutein concentration of 50 *μ*g/mL (v/v) was chosen after performing pilot experiments comparing different concentrations (0.5 *μ*g/mL–50 *μ*g/mL, data not shown).

### 2.3. Enzyme-Linked Immunosorbent Assay

Supernatants from mature adipocytes cultured with or without lutein were collected after 24 and 48 hours and stored at −80°C for FD measurements. The secretion level of FD was measured at a 1/100 dilution by using a commercially available development kit (DuoSet) for human complement factor D (R&D Systems, Minneapolis, USA) according to the manufacturer's instructions.

### 2.4. Quantitative Real-Time PCR (qRT-PCR)

The expression of FD mRNA was assessed by quantitative real-time PCR (qRT-PCR). Total RNA was isolated from the mature adipocytes incubated with or without lutein for 48 hours by using the RNeasy Mini Kit (Qiagen Westburg, Leusden, The Netherlands) according to the manufacturer's protocol. The quantity and purity of the mRNA were measured by using the NanoDrop system (Thermo Scientific, USA). 500 ng of each RNA sample was reverse transcribed for the first-strand cDNA by using the* iScript* cDNA synthesis kit protocol (Bio-Rad Laboratories B.V., The Netherlands) and then diluted 40 times with distilled water. The following primers were purchased from Operon (Sigma-Aldrich, The Netherlands) and used for real-time PCR (FD forward 5′-GTCCTGGTGGCGGAGC-3′, reverse 5′-AGAACCTGCACCTTCCCGTC-3′: *β*-actin forward 5′-GACTACCTCATGAAGATCCT-3′, reverse 5′-GCGGATGTCCACGTCACACT-3′). The mixture reaction contained 12.5 *μ*L SYBR^©^ Green Supermix (Bio-Rad), 5 *μ*L diluted cDNA, and 0.3 *μ*M primers in a total volume of 25 *μ*L. The two-step qRT-PCR was performed under the following cycling conditions which consisted of an initial denaturation at 95°C for 3 min, followed by 40 alternating cycles of 95°C for 10 sec and 55°C for 45 sec, respectively. All PCR reactions included a cDNA dilution curve to assess PCR efficiency and all reactions were followed by a melt curve (55–95°C). Data were analyzed by using MyiQ Software system (Bio-Rad) and the amount of target cDNA in each sample was determined by a fractional PCR threshold cycle number (Ct-value) and compared to the corresponding Ct-value for the housekeeping gene *β*-actin. The relative gene expression level for each gene was calculated by using the 2-Delta Delta C(T) method [52].

### 2.5. Statistical Analysis

Statistical analysis was performed using SPSS 20.0.0; one-way analysis of variance (ANOVA) with Post hoc Bonferroni test was used for data analysis. All values are expressed as mean ± standard error of the mean (SEM). A value of *p* < 0.05 was considered statistically significant.

## 3. Results

### 3.1. The Differentiation Process of SGBS Adipocytes

**Figure 1:**
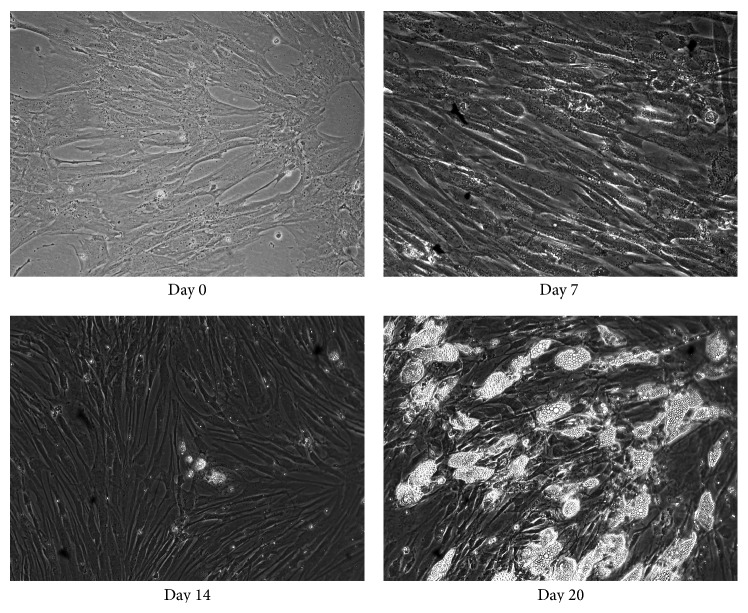
The differentiation process of SGBS preadipocytes to mature adipocytes at days 0, 7, 14, and 20.

SGBS preadipocytes were differentiated into mature adipocytes during 20 days. Lipid accumulation became visible after 7 days and increased further during the differentiation period (Figure 1). After 20 days, approximately 50–60% of the cells were fully differentiated as demonstrated by the massive triglyceride accumulation.

### 3.2. The Effect of Lutein on FD Secretion by Mature Adipocytes

**Figure 2:**
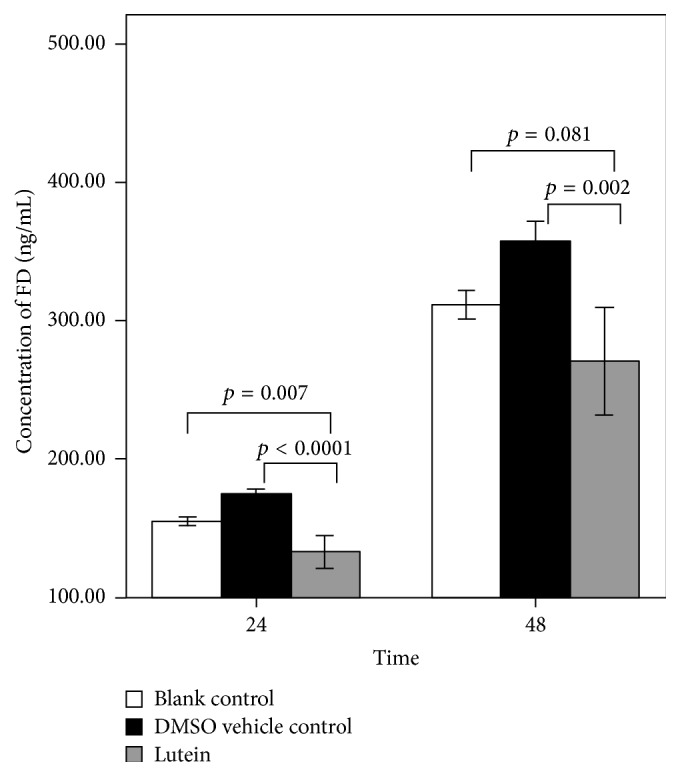
Factor D (FD) concentration in culture medium from 20-day differentiated SGBS adipocytes incubated for 24 h and 48 h in culture medium (blank control), 0.5% DMSO (DMSO vehicle control), or lutein (50 *μ*g/mL) in the presence of 0.5% DMSO (lutein).

Lutein was added to mature adipocyte cultures and the mean FD concentration in the culture medium was measured at 24 h and 48 h. As control, adipocyte cultures that contain the similar medium with DMSO only (vehicle control) or cells with medium only (blank group) were used (Figure 2). The mean FD concentration increased with time in the blank group from 155.3 ± 3.1 ng/mL at 24 h to 311.8 ± 10.2 ng/mL at 48 h. In the DMSO vehicle control group the mean FD concentration increased from 174.1 ± 4.3 ng/mL to 357.6 ± 14.5 ng/mL between the 24 h and 48 h time points, respectively. Addition of lutein to the cultures resulted in a decreased concentration of FD as compared to the two control groups. Particularly, a significantly decreased FD level was observed when comparing the lutein group with the vehicle control group: 174.1 ± 4.3 ng/mL (DMSO control) versus 133.3 ± 11.9 ng/mL (lutein) (*p* < 0.0001) at 24 h and 357.6 ± 14.5 ng/mL (DMSO control) versus 271.1 ± 38.7 ng/mL (lutein) (*p* = 0.002) at 48 h. When comparing the blank control with the DMSO vehicle control, it is clear that DMSO resulted in a slight increase in the FD production by the adipocytes: 155.3 ± 3.1 ng/mL (blank control) versus 174.1 ± 4.3 ng/mL (DMSO control) at 24 h (*p* = 0.017) and 311.8 ± 10.2 ng/mL (blank control) versus 357.6 ± 14.5 ng/mL (DMSO control) at 48 h (*p* = 0.081).

### 3.3. Lutein Downregulated the mRNA Expression of FD in Adipocytes

**Figure 3:**
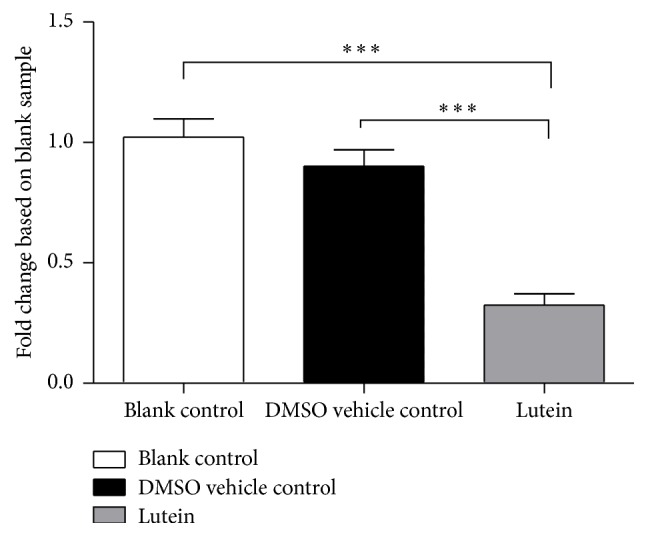
Factor D mRNA expression in 20-day differentiated SGBS adipocytes incubated for 48 h with culture medium (blank control), 0.5% DMSO (DMSO vehicle control), or lutein (50 *μ*g/mL) in the presence of 0.5% DMSO (lutein). Bars represent fold change of FD mRNA expression compared to blank control (^*∗∗∗*^ *p* < 0.0001).

The aforementioned results showed that lutein inhibited FD protein secretion. To examine whether lutein also affects FD mRNA expression in adipocytes we harvested 20-day differentiated SGBS adipocytes that were incubated for an additional 48 h with lutein. We performed a qPCR analysis whereby FD mRNA expression was measured relative to *β*-actin mRNA (household gene). The results showed that incubation of mature SGBS adipocytes with lutein for 48 hours significantly downregulated FD mRNA expression when compared to both the blank control and the vehicle control (DMSO) (*p* < 0.0001 and *p* < 0.0001, resp.) (Figure 3). No difference was observed in the mRNA expression between the DMSO group and the blank group (*p* = 0.37).

## 4. Discussion

In this study we show that lutein suppresses factor D (FD) expression in mature SGBS adipocytes, both at the level of protein secretion and at the mRNA level. To the best of our knowledge, this is the first study to examine the influence of lutein on the expression of FD in human adipocytes. Adding lutein to SGBS adipocyte cultures resulted in a 23% reduction at 24 hours and a 24% reduction at 48 hours of the release of FD as compared to vehicle controls. Adipose tissue is the main source of FD [46] and earlier data using SGBS adipocytes already showed that these cells were able to secrete FD [53]. At the same time, adipose tissue also serves as the main storage site for carotenoids such as lutein and these facts prompted us to study a possible interaction between these two factors [54]. The* in vitro* observations from this study support our earlier* in vivo* findings showing that taking a lutein supplement reduces circulating FD levels [42].

How lutein is taken up by adipocytes is not clear yet. Lutein biosynthesis only occurs in plants, algae, bacteria, and certain fungi. Humans cannot make lutein and uptake is dependent on the dietary intake of certain fruits, leafy vegetables, or eggs [55]. In the gut, lutein is taken up by enterocytes, packed into chylomicrons, and then transported via lymphatics, thoracic duct, and bloodstream to the liver hepatocytes [56]. In the hepatocytes it is bound to lipoproteins and then transported via the blood to various sites in the body including the retina and adipose tissue [57]. Body fat is an important storage site for lutein and it may compete with other tissues, thereby making it less available for the macula [58]. Possible differences in adipose tissue composition and distribution may explain differences observed in lutein metabolism between men and women [59]. Both lutein and factor D have separately been implicated in the pathogenesis of AMD and our observation linking these two factors is a novel observation [60].

The presence of FD in adipose tissue has been attributed to its role in the local cleavage of complement component C3, thereby forming C3a [61]. The carboxy-terminal arginine of C3a is subsequently cleaved by carboxypeptidase N to generate C3a-desarg, also known as acylation stimulating protein (ASP) [62]. ASP interacts with the ASP receptor (C5L2) on adipocytes, thereby triggering triglyceride synthesis [62].

How FD expression is regulated in adipose tissue did not receive much attention yet. FD mRNA expression is increased during the differentiation of preadipocytes to adipocytes [63]. In addition, FD secretion was measured in the medium of SGBS adipocytes but not in the medium of SGBS preadipocytes [53]. On the other hand,* in vitro* stimulation with retinoic acid (RA) resulted in a 4-5-fold suppression of FD mRNA expression by mouse adipocytes [64].

Under normal conditions an adipocyte will already have a lutein store inside the cells [59]. The amount of lutein in adipose tissue ranges between 0.09 Mol (men) and 0.36 Mol (women) [59], which is a few thousandfold higher than the dose (50 *μ*g/mL is equivalent to 87 *μ*Mol) used in our study to load the cells. The SGBS cells we used were cultured under lutein-free conditions and will thus not yet have acquired lutein inside the cells. In our experiments we cultured the SGBS cells for up to 48 hours with lutein with a dose that is much higher than that found in human plasma (0.22 *μ*Mol) [25]. Using this short time period we found that lower doses than the 50 *μ*g/mL did not have an effect. Further experiments should be done using both longer and shorter time intervals and with varying doses of lutein to investigate how different adipose lutein levels might affect FD expression in our* in vitro* model. As mentioned above, an adipocyte in the human body already contains a certain concentration of lutein and we believe that the level of lutein in an adipocyte will control the steady-state production of FD. Future experiments with adipose tissue taken from humans whereby lutein content is correlated with FD levels will provide evidence to show whether this hypothesis is correct.

The blood level of ASP (C3a-desarg) has been shown to be increased in patients with AMD and this has added support to the hypothesis that AMD is a disease caused by a hyperactive alternative complement pathway [65]. FD levels have also been found to be increased in the blood of AMD patients [36, 37]. As mentioned earlier, FD is the rate limiting enzyme of the alternative pathway and small changes in its concentration can potentially have profound effects on the generation of biologically active products such as C3a, C3a-desarg, and C5a.

Obesity has been shown to be a risk factor for AMD but the exact role of adipose tissue in the pathogenesis of AMD is not yet clear [7, 66]. Plasma levels of FD are associated with body mass index (BMI) and were shown to be higher in obese versus nonobese subjects [67]. The evidence shown above suggests a possible role for adipocyte biology in AMD pathogenesis [60].

A role for FD in retinal disease became apparent from an experimental mouse model showing that photoreceptors were protected from light induced damage in FD knockout animals [68]. Control of FD has now been brought to the clinic with the availability of a humanized IgG Fab murine anti-factor D antibody (FCFD4514S) that has been shown to block the formation of the alternative pathway C3 convertase [69]. The FD antibody was named Lampalizumab, and phase 1 and phase 2 trials, whereby it was given intravitreally in patients with geographic atrophy, have shown promising results [70] and are now followed by two phase-3 trials (these trials are registered with https://clinicaltrials.gov/).

In conclusion, we have shown that lutein suppresses FD expression in and secretion by human SGBS adipocytes. This observation may explain the decrease in circulating FD with daily lutein supplementation that we observed in an earlier study [42]. It might offer a novel therapeutic approach to prevent the progression of AMD and other inflammatory diseases that are modulated by FD.
